# Medical ethnobotany

**DOI:** 10.1186/s12906-024-04515-0

**Published:** 2024-06-05

**Authors:** Adeyemi O. Aremu, Binsheng Luo, Sakina Mussarat

**Affiliations:** 1 School of Life Sciences, College of Agriculture, Engineering and Science, University of KwaZulu-Natal, Private Bag X54001, Durban, 4000 South Africa; 2 Indigenous Knowledge Systems Centre, Faculty of Natural and Agricultural Sciences, North-West University, Private Bag X2046, Mmabatho, 2735 South Africa; 3 Lushan Botanical Garden, Jiangxi Province and Chinese Academy of Sciences, Lushan, 332900 China; 4 Department of Botany, Kohat University of Science and Technology, Kohat, 26000 Pakistan

**Keywords:** Antimicrobial, Conservation, Ethnopharmacology, Indigenous knowledge, Medicinal plants, Natural products, Phytomedicine

## Abstract

This collection on medical ethnobotany focuses on contributions that explore the invaluable potential associated with the ethnobotanical uses of medicinal plants, their phytochemical profiling, safety, and efficacy studies as well as their cultural and ecological context. This call for papers is expected to expand the knowledge base on how medicinal plants contribute toward the achievement of the United Nations Sustainable Development Goals (UN SDGs), in this case, goal 15 (life on land).

## Introduction

From time immemorial, the therapeutic value of plants and their effectiveness in the treatment and management of the health and general well-being of humans have been recognized among different ethnic groups. Particularly, plants are known to play a vital role in ancient medicinal systems such as the African, Ayurvedic and Chinese medicines. In recent time, the acceptance of traditional medicine has increased, which is often attributed to their accessibility and affordability especially in countries with larger populations.

In modern medicine, plants are recognized sources of important drugs such as artemisinin, ginkgolides, quinine, reserpine, scopolamine, paclitaxel, vincristine and vinblastine. It is generally acknowledged that natural resources including plants continue to influence the design of molecules that are developed into useful drugs as remedies for several diseases (e.g., cancer, malaria, and dementia) affecting humans [1, 2]. Recently, there is a renewed scientific interest in plant-derived natural product-based drug discovery to manage the ever-challenging global health needs in the 21st century [3, 4]. In an attempt to apply a transdisciplinary approach to medical ethnobotany, it is essential to establish the link between the ecosystems and human societies that is facilitated by the relationship existing between traditional/indigenous knowledge and their sustainability as well as the equitable use of medicinal plants [4]. It is envisaged that this approach will serves as a vital tool, resonating as a viable means to address and achieve the United Nations Sustainable Goals (UN SDGs) especially ‘’life on land’’ (UN SDG goal 15).

Globally, we are witnessing an increasing loss of biodiversity and associated indigenous knowledge [5]. This problem is attributed to climate change and anthropogenic activities especially the unsustainable harvesting of plant resources [4, 6]. The need for a fine balance between the use of wild medicinal plant populations and their conservation cannot be overemphasized. From an ecological perspective, indiscriminate harvesting of medicinal plant populations below the sustainable threshold results in the irreversible reduction of reproductive capacity which significantly affects the conservation status of the medicinal plant species overtime [5, 7, 8]. Furthermore, the continuous degradation of ecosystems due to human activities (e.g., agriculture, urbanization, mining) has been identified as a major driver of emerging infectious diseases and zoonotic outbreaks [5]. Another often neglected aspect is the unintended valuable medical knowledge on plants that is linked to the increasing language extinction especially among the often neglected indigenous communities across the world [9]. The transmission and sharing of indigenous knowledge has primarily been through verbal communication, thereby increasing the degree of loss of these knowledge systems through successive generations. This is a further justification for the continuous effort aimed at the documentation of medicinal plants and their uses. This also resonates with one of the action points in the Shenzhen declaration that addresses the need to value, document, and protect indigenous, traditional, and local knowledge about plants and nature [6]. After documenting the uses of plants, generating evidence on their biological efficacies, safety and phytochemicals are pertinent [10, 11], thereby improving natural product research translation: ‘’from source to clinical trial’’.

### Call for the collection series

#### About the collection series

BMC Complementary Medicine and Therapies has launched this collection in support of the United Nations Sustainable Development Goals (UN SDGs), specifically Goal 15 “Life on Land” and aims to explore the significance of medical ethnobotany within the field of complementary medicine, and to underline the importance of conserving and respecting indigenous knowledge.

We welcome studies that identify, document, and study the ways in which communities and people use medicinal plants, research into their chemical compounds, including their safety and efficacy, and research into the cultural and ecological context of plants as medicine. Submissions to this collection should follow the guidelines provided by BMC Complementary Medicine and Therapies. Manuscripts will undergo a peer-review process to ensure the highest scientific quality and relevance to the theme of the collection.

## Data Availability

No datasets were generated or analysed during the current study.
